# ﻿A new loach species of the genus *Oreonectes* (Teleostei, Cypriniformes, Nemacheilidae) from Guangxi, China

**DOI:** 10.3897/zookeys.1196.109810

**Published:** 2024-03-28

**Authors:** Xue-Ming Luo, Rui-Gang Yang, Li-Na Du, Fu-Guang Luo

**Affiliations:** 1 Key Laboratory of Ecology of Rare and Endangered Species and Environmental Protection (Guangxi Normal University), Ministry of Education Guilin 541004, China; 2 Guangxi Key Laboratory of Rare and Endangered Animal Ecology, College of Life Science, Guangxi Normal University, Guilin 541004, China; 3 Scientific Research Academy of Guangxi Environmental Protection, Nanning, Guangxi 530022, China; 4 Liuzhou Aquaculture Technology Extending Station, Liuzhou 545006, China

**Keywords:** New species, morphology, phylogeny, taxonomy, Xijiang River

## Abstract

A new loach species, *Oreonectesandongensis***sp. nov.** is described from the Guangxi Zhuang Autonomous Region, China. The new species can be differentiated from other members of the genus by combinations of characters: a developed posterior chamber of the swim bladder, 13–14 branched caudal-fin rays, 8–16 lateral-line pores, body width 12–15% of standard length (SL), interorbital width 42–47% of head length (HL), and caudal peduncle length 11–16% of SL. Bayesian inference phylogenetic analysis based on mitochondrial Cyt *b* provided strong support for validity of *O.andongensis***sp. nov.** (uncorrected *p*-distance 6.0–7.5%).

## ﻿Introduction

The freshwater fish genus *Oreonectes* Günther, 1868, which belongs to the family Nemacheilidae, exhibits notable adaptations to the karst geomorphic environment. These small fish are predominantly distributed in southern China (Xijiang River system of Guangxi Zhuang Autonomous Region, Pearl River system of Guangdong Province, and Pearl River system of Hong Kong) and Kalong River of northern Vietnam (Quảng Ninh Province in northeast Vietnam) (Kottelat 2012). The genus was first established by Günther in 1868, designating *Oreonectesplatycephalus* Günther, 1868 as the type species, collected from a small stream in Hong Kong within the Pearl River system. The diagnosis of the genus included a slightly compresses body, a markedly depressed head, and the origin of the dorsal fin much closer to the base of the caudal fin than to the operculum Günther (1868). Later, till 2006 a number of species have been added to the genus: *O.anophthalmus* Zheng, 1981 (an underground river in Taiji Cave, Wuming County, Guangxi, Youjiang River, China), *O.furcocaudalis* Zhu & Cao, 1987 (a subterranean water outlet in the suburbs of Rongshui County, Guangxi, Liujiang River, China), *O.retrodorsalis* Lan, Yang & Chen, 1995 (an underground river outlet in Longli Village, Nandan County, Guangxi, Hongshui River, China), and *O.translucens* Zhang, Zhao & Zhang, 2006 (Xia’ao Town, Du’an County, Guangxi, Hongshui River, China) (Zheng 1981; Zhu 1989; Lan et al. 1995; Zhang et al. 2006). Subsequently, a revision of *Oreonectes* was published with description of two new species *O.microphthalmus* Du, Chen & Yang, 2008 (Du’an County, Guangxi, Hongshui River, China) and *O.polystigmus* Du, Chen & Yang, 2008 (Dabu Village, Guilin City, Guangxi, Lijiang River, China) (Du et al. 2008). Meanwhile, Du et al. (2008) subdivided the genus into two groups based on the caudal fin morphology: the round caudal fin group (*O.platycephalus* group), containing *O.anophthalmus*, *O.platycephalus*, *O.polystigmus*, and *O.retrodorsalis*, and the forked caudal fin group (*O.furcocaudalis* group), including *O.furcocaudalis* and *O.microphthalmus*. After 2009, various species were described, including *O.macrolepis* Huang, Du, Chen & Yang, 2009 (an underground river in Dacai Town, Huanjiang County, Guangxi, Xijiang River system, China), *O.luochengensis* Yang, Wu, Wei & Yang, 2011 (a cave near Tianhe Town, Luocheng County, Guangxi, Xijiang River system, China), *O.guananensis* Yang, Wei, Lan & Yang, 2011 (an underground karst cave outlet near Guan’an Village, Changmei Town, Huanjiang County, Guangxi, Xijiang River system, China), *O.elongatus* Tang, Zhao & Zhang, 2012 (Mulun Town, Huanjiang County, Guangxi, Longjiang River, China), *O.acridorsalis* Lan, 2013 (a cave near Bamu Town, Tian’e County, Guangxi, Hongshui River, China), *O.barbatus* Gan, 2013 (first described from a cave near Lihu Town, Nandan County, Guangxi, Hongshui River, China), *O.donglanensis* Wu, 2013 (a cave near Simeng Town, Donglan County, Guangxi, Hongshui River, China), *O.duanensis* Lan, 2013 (a cave near Chengjiang Town, Du’an County, Guangxi, Hongshui River, China), *O.daqikongensis* Deng, Xiao, Hou & Zhou, 2016 (Seven Big Scenic Spot, Libo County, Guizhou, Hongshui River, China), *O.shuilongensis* Deng, Wen, Xiao & Zhou, 2016 (a cave near Shuilong Town, Sandu County, Guizhou, Duliu River, China), *O.guilinensis* Huang, Yang, Wu & Zhao, 2020 (Shigumen Village, Xingping Town, Yangshuo County, Guilin City, Guangxi, Lijiang River, China), and *O.damingshanensis* Yu, Luo, Lan, Xiao & Zhou, 2023 (Waminggu Scenic Area, Leping Village, Guling Town, Mashan County, Guangxi, Xijiang River system, China) (Huang et al. 2009, 2020; Yang et al. 2011a, b; Tang et al. 2012; Lan et al. 2013; Deng et al. 2016a, b; Yu et al. 2023). Zhang et al. (2016) established the genus *Troglonectes* Zhang, Zhao & Tang, 2016, designating *O.furcocaudalis* as the type species, with *O.barbatus*, *O.elongatus*, *O.macrolepis*, *O.microphthalmus*, and *O.translucens*, characterized by a forked caudal fin, a developed adipose crest of the caudal fin, and the dorsal-fin origin above the pelvic-fin origin. Later studies added *O.daqikongensis*, *O.donglanensis*, *O.duanensis*, *O.retrodorsalis*, and *O.shuilongensis* to *Troglonectes* (Huang et al. 2020; Du et al. 2023), while *O.anophthalmus* and *O.acridorsalis* were assigned to a new genus, *Karstsinnectes* Zhou, Luo, Wang, Zhou & Xiao, 2023 based on morphological and molecular evidence (Luo et al. 2023).

Until now, the genus of *Oreonectes* contains six valid species, namely, *O.damingshanensis*, *O.guananensis*, *O.guilinensis*, *O.luochengensis*. *O.platycephalus*, and *O.polystigmus*. In July 2022, ten loach specimens were collected from Laibin City in the Hongshui River system, Guangxi Zhuang Autonomous Region, China. Morphological features and molecular data suggest that the specimens under consideration represent a previously undescribed species within the genus *Oreonectes*, which are described herein.

## ﻿Materials and methods

Field collections followed the Guide to Collection, Preservation, Identification, and Information Share of Animal Specimens (Xue 2010) and Implementation Rules of Fisheries Law of the People’s Republic of China. All activities followed the Laboratory Animal Guidelines for the Ethical Review of Animal Welfare (GB/T 35892–2018). Specimens of the new species were collected by FGL. Samples were collected using a hand net and mesh traps. Freshly caught fish were euthanized using eugenol. After death, the pectoral fins from the right side were taken and preserved in ethanol for molecular analysis. Specimens used for morphological studies were preserved in 10% formalin, before being transferred to 75% ethanol for long-term storage at the collection of the Key Laboratory of Ecology of Rare and Endangered Species and Environmental Protection (Guangxi Normal University), Ministry of Education.

### ﻿Phylogenetic analyses

Mitochondrial cytochrome *b* gene (Cyt *b*) sequences were sequenced by the Science Corporation of Gene (China) following standard Illumina protocols. Genome sequencing data were submitted to GenBank (Accession No. OR188128, OR712240, OR712241). We retrieved 36 Cyt *b* sequences of Nemacheilidae species from the NCBI GenBank database (Appendix 1) for phylogenetic tree reconstruction to test the phylogenetic positions of *Oreonectesandongensis* sp. nov. All sequences were aligned in MEGA v. 11.0 (Tamura et al. 2021) by the MUSCLE (Edgar 2004) algorithm with default parameters, and then sequences were executed in PartitionFinder v. 2.1.1 (Lanfear et al. 2017) in order to select the most appropriate model of evolution to be used for phylogenetic analyses. The model selection for each codon position of the complete mitochondrial genes indicated that the best fit models were K80+I+G for the first codon, HKY+I+G for the second codon, and TRN+I+G for the third codon. Bayesian inference (BI) analysis was performed using MRBAYES v. 3.2.6 (Ronquist et al. 2012). The chains were run for two million generations and sampled every 1000 generations. The first 25% of the sampled tree was discarded as burn-in, and the remaining trees were used to create a consensus tree and to estimate Bayesian posterior probabilities (BPP). Nodes in the trees were considered well-supported at BPP ≥ 0.95.

### ﻿Morphological examination

Methods used for counts and measurements followed Du et al. (2021) and characteristics of the cephalic lateral line system were examined following Kottelat (1990) and Tang et al. (2012). All measurements were taken point-to-point with dial calipers to the nearest 0.1 mm. Abbreviations used in the text are as follows: **AFBL** for anal-fin base length, **AFL** for anal-fin length, **BD** for body depth, **BW** for body width, **CPL** for caudal peduncle length, **CPD** for caudal peduncle depth, **DAPN** for distance between anterior and posterior nostrils, **DAN** for distance between anterior nostrils, **DFBL** for dorsal-fin base length, **DFL** for dorsal-fin length, **DPN** for distance between posterior nostrils, **ED** for eye diameter, **HD** for head depth, **HL** for head length, **HW** for head width, **ISBL** for inrostral barbel length, **IW** for interorbital width, **MBL** for maxillary barbel length, **OSBL** for outrostral barbel length, **PANL** for preanus length, **PAL** for preanal length, **PDL** for predorsal length, **PFBL** for pectoral-fin base length, **PFL** for pectoral-fin length, **PPL** for prepectoral length, **PVL** for prepelvic length, **STL** for snout length, **SL** for standard length, **TL** for total length, **VFBL** for pelvic-fin base length, and **VFL** for pelvic-fin length.

## ﻿Results

### ﻿Genetic evidence from phylogenetic analysis

**Figure 1.**
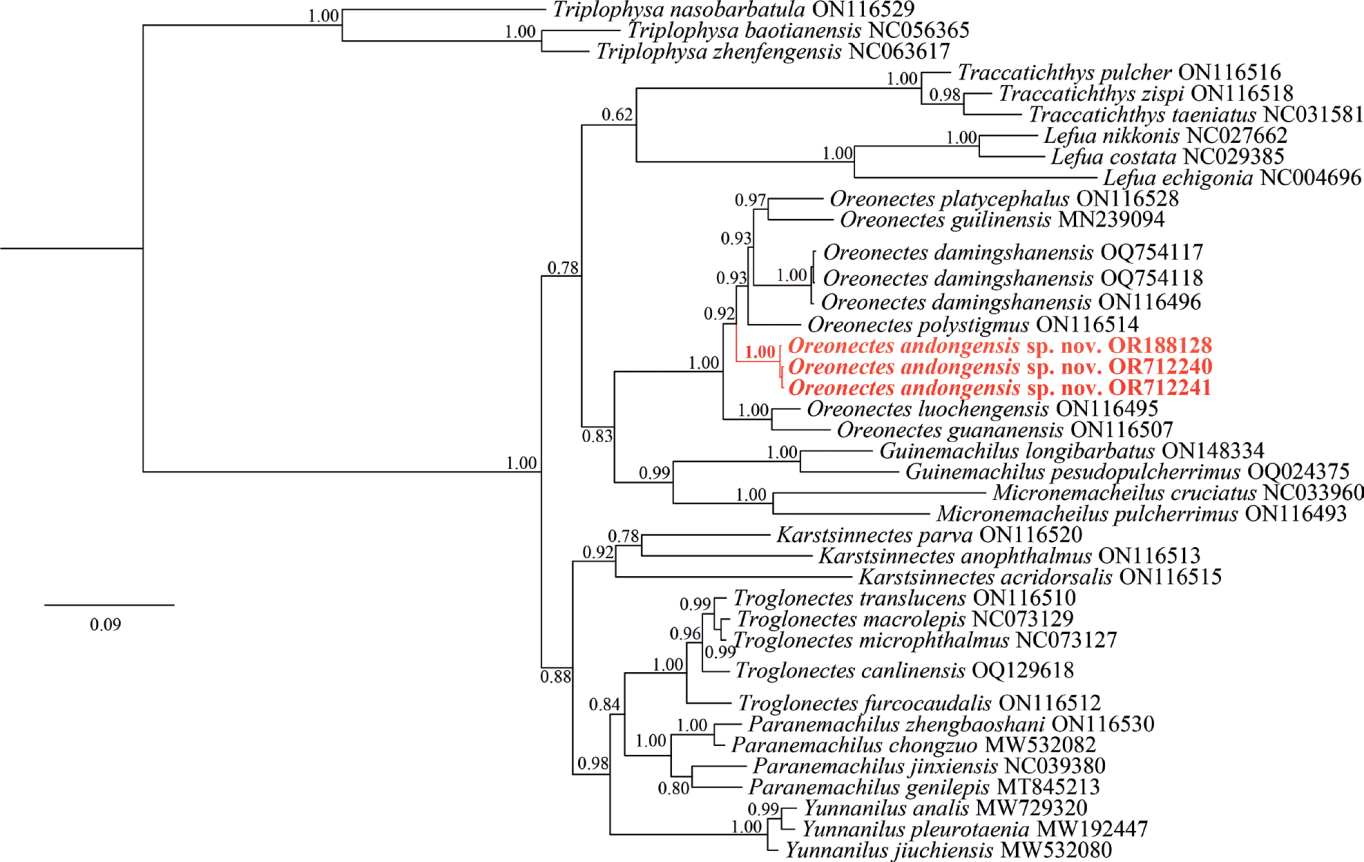
Bayesian phylogenetic tree of *Oreonectes* based on mitochondrial Cyt *b.* Numbers above branches are BPPs.

BI analyses were performed to construct a phylogenetic tree, revealing consistent topologies based on Cyt *b* sequences spanning 1141 bp. The phylogenetic tree affirmed the validity of the new species with high nodal support (BPP ≥ 0.95). Additionally, members of the Oreonectes genus constituted a monophyletic group, which was phylogenetically sister to the *Guinemachilus*Du et al., 2023 and *Micronemacheilus* clade (Fig. 1). *Oreonectesandongensis* sp. nov. formed a highly supported clade with *O.damingshanensis*, *O.guilinensis*, *O.platycephalus*, and *O.polystigmus*.

The uncorrected *p*-distances of Cyt *b* between *Oreonectesandongensis* sp. nov. and the other six species ranged from 6.0% (for *O.polystigmus*) to 7.5% (for *O.guananensis*) (Table 2).

### ﻿Taxonomy

#### ﻿ Oreonectes andongensis

Taxon classificationAnimaliaCypriniformesNemacheilidae

Luo, Yang, Du & Luo sp. nov.

BFECA430-0E51-5032-9598-614CC3D910AB

https://zoobank.org/31E2362E-71AF-4E1F-B78A-53DFB1980F33

**Figure 2.**
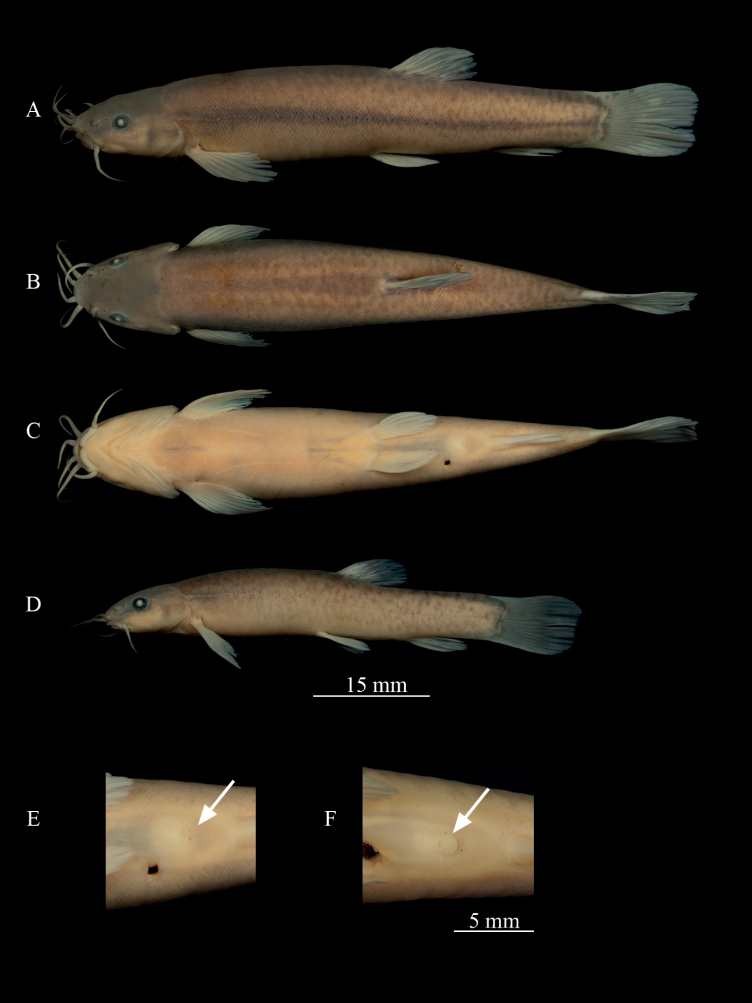
*Oreonectesandongensis* sp. nov. **A–C** lateral, dorsal and ventral views of holotype GXNU20220601 (♀), **D** lateral view of paratype GXNU20220610 (♂), **E, F** gonadal structure of female (**E**) and male (**F**).

**Figure 3.**
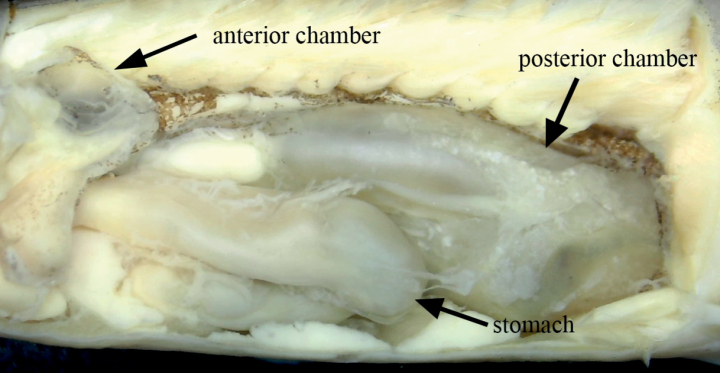
Stomach, anterior chamber, and posterior chamber of *Oreonectesandongensis* sp. nov.

**Figure 4.**
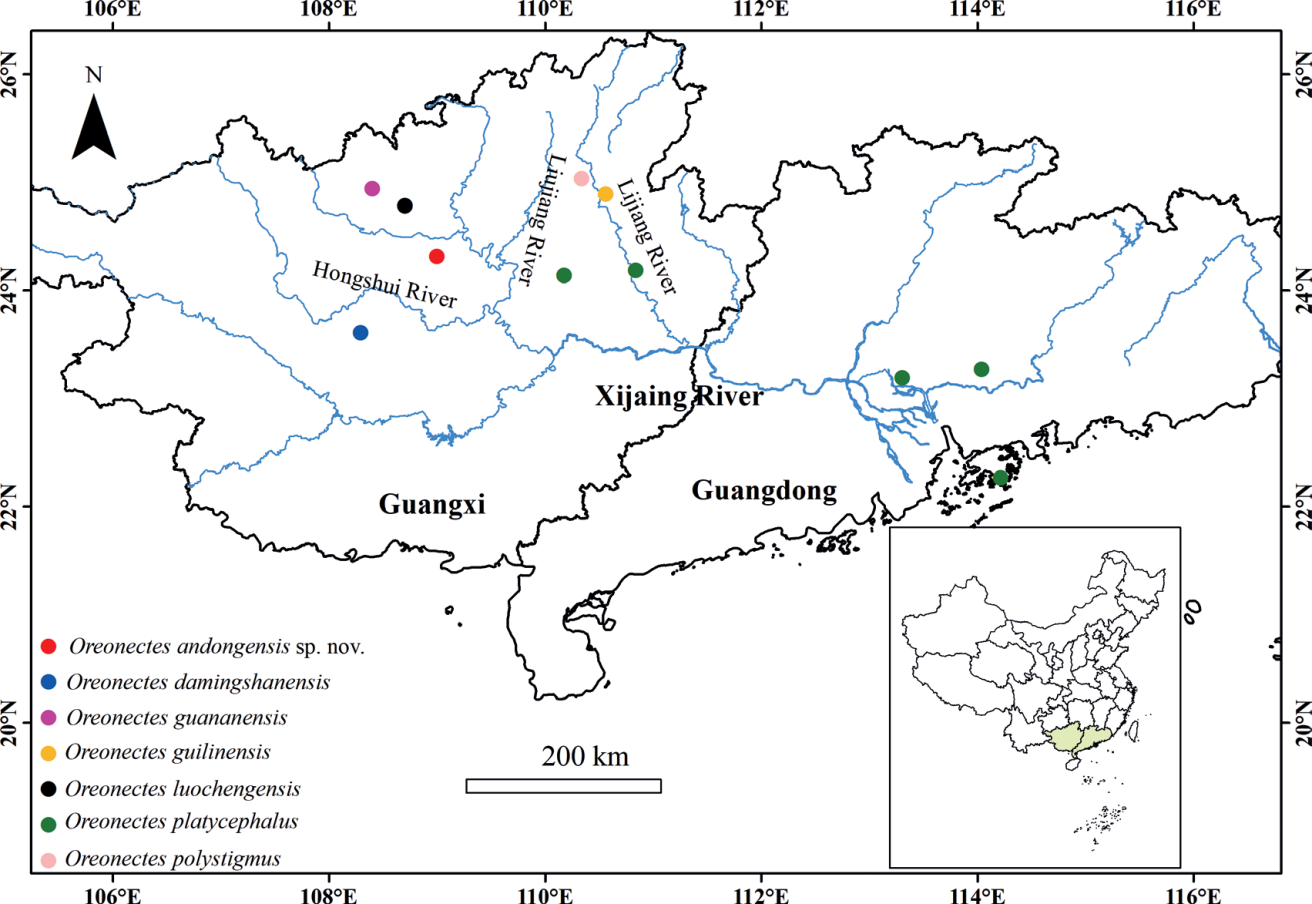
Distribution of *Oreonectes* in southern China.

**Figure 5.**
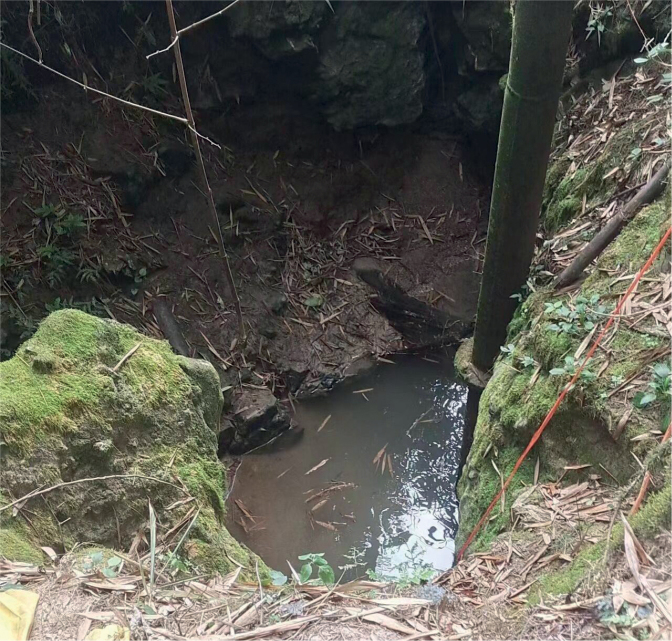
Habitat of *Oreonectesandongensis* sp. nov.

Table 1 , Figs 1 , 2 , 3 , 4 , 5

##### Type material.

***Holotype*.** GXNU20220601, 74.9 mm standard length (SL), Andong Town, Xincheng County, Laibin City, Hongshui River system, Guangxi Zhuang Autonomous Region, China, 24°18.57'N, 108°59.61'E, 179 m a.s.l., collected by F.G.L., 20 July 2022. ***Paratypes*.** GXNU20220602–10, 9 specimens, 45.9–68.7 mm SL, same data as holotype.

##### Diagnosis.

The new species is assigned to the genus *Oreonectes* based on Cyt *b* phylogenetic analysis and morphological characters. The new species can be distinguished from other members of *Oreonectes* by the following combination of characters: posterior chamber of swim bladder developed (vs reduced in *O.platycephalus*), color pattern present (vs colorless in *O.luochengensis*), tip of pelvic fin not reaching anus (vs exceeding anus in *O.polystigmus* and *O.guilinensis*), dorsal-fin origin slightly posterior to pelvic-fin origin (vs opposite in *O.guananensis*), six branched pelvic-fin rays (vs 7 or 8 in *O.damingshanensis*, *O.guananensis*, *O.luochengensis*). *Oreonectesandongensis* sp. nov. can be further differentiated from *O.damingshanensis* by more numerous, better developed inner gill rakers on the first gill arch (11–12 vs 9).

##### Description.

The morphometric data of the holotype and paratypes are in Table 1. Three unbranched and seven branched dorsal-fin rays, one unbranched and nine or ten branched pectoral-fin rays, one unbranched and six branched pelvic-fin rays, three unbranched and five branched anal-fin rays, 13 or 14 branched caudal-fin rays, and 11 or 12 inner-gill rakers on first gill arch (in 3 specimens).

**Table 1.** Morphological data and habitat types of the genus *Oreonectes*. Data of *O.damingshanensis* is from the original description (Yu et al. 2023).

	*O.andongensis* sp. nov.	*O.damingshanensis*	*O.guananensis*	*O.guilinensis*	*O.luochengensis*	*O.platycephalus*	*O.polystigmus*
	Range (Mean ± SD)	Range (Mean ± SD)	Range (Mean ± SD)	Range (Mean ± SD)	Range (Mean ± SD)	Range (Mean ± SD)	Range (Mean ± SD)
TL (mm)	45.9–74.9 (57.1 ± 9.9)	63.7–98.9 (78.8 ± 8.8)	62.9–90.2 (75.7 ± 10.5)	63.2–89.7 (80.4 ± 7.6)	77.2–92.1 (84.2 ± 4.8)	49.6–82.5 (65.2 ± 11.7)	41.8–67.8 (52.2 ± 8.0)
SL (mm)	36.5–60.2 (46.2 ± 8.4)	52.5–81.8 (64.9 ± 7.3)	51.0–71.5 (60.9 ± 8.3)	52.0–73.5 (65.9 ± 6.1)	62.2–74.7 (68.1 ± 4.3)	38.7–64.2 (51.2 ± 9.3)	33.7–54.7 (42.5 ± 6.7)
Percentage of SL (%)							
BD	15.7–17.9 (16.7 ± 0.7)	14.2–18.1 (15.5 ± 1.3)	16.2–19.6 (17.6 ± 1.3)	16.7–18.5 (18.0 ± 0.5)	16.0–18.3 (17.3 ± 0.7)	14.8–19.3 (17.2 ± 1.4)	12.5–19.5 (16.3 ± 2.2)
BW	11.5–15.2 (12.7 ± 1.0)	10.4–12.5 (11.3 ± 0.6)	9.5–13.1 (11.3 ± 1.3)	12.7–14.8 (13.9 ± 0.6)	9.8–12.1 (10.8 ± 0.8)	9.5–11.5 (10.5 ± 0.5)	7.4–11.8 (8.7 ± 1.2)
HW	14.7–17.2 (15.7 ± 0.7)	14.4–17.8 (16.1 ± 1.0)	14.8–16.9 (15.9 ± 0.7)	15.4–19.0 (17.0 ± 1.1)	13.7–16.9 (14.9 ± 0.9)	14.6–17.5 (15.7 ± 0.8)	13.7–17.5 (15.5 ± 1.2)
HD	11.4–12.8 (12.1 ± 0.5)	10.8–12.9 (12.0 ± 0.7)	10.9–11.9 (11.4 ± 0.4)	12.0–15.2 (13.3 ± 1.0)	10.0–12.4 (11.1 ± 0.7)	10.3–13.5 (11.3 ± 0.9)	11.1–13.1 (12.1 ± 0.7)
HL	21.1–24.5 (22.3 ± 0.9)	20.2–23.0 (21.7 ± 0.9)	21.8–23.2 (22.6 ± 0.5)	20.3–24.2 (21.7 ± 1.1)	20.7–24.2 (22.4 ± 1.0)	20.8–25.4 (22.8 ± 1.4)	22.3–24.5 (23.6 ± 0.6)
PDL	57.7–62.4 (60.0 ± 1.7)	59.5–62.4 (60.7 ± 0.8)	57.2–59.5 (58.4 ± 0.8)	57.4–60.4 (58.9 ± 0.9)	58.0–63.3 (60.0 ± 1.5)	60.0–64.7 (62.0 ± 1.9)	59.2–63.9 (60.9 ± 1.6)
DFL	16.8–19.2 (18.1 ± 0.7)	17.2–21.7 (18.9 ± 1.2)	17.6–18.6 (18.0 ± 0.3)	16.6–19.0 (17.4 ± 0.8)	16.9–19.2 (18.2 ± 0.7)	15.7–21.1 (19.4 ± 1.6)	16.8–19.8 (18.1 ± 1.0)
DFBL	8.9–11.6 (10.4 ± 0.8)	9.0–11.3 (10.3 ± 0.7)	9.5–11.0 (10.1 ± 0.6)	7.2–10.4 (8.8 ± 0.9)	6.6–7.3 (7.0 ± 0.3)	9.4–11.1 (10.1 ± 0.5)	8.9–12.1 (10.0 ± 1.0)
PPL	19.4–23.7 (21.2 ± 1.2)	19.1–22.7 (21.4 ± 1.0)	21.4–23.0 (22.0 ± 0.5)	16.9–20.4 (19.1 ± 1.2)	19.0–21.8 (20.3 ± 0.7)	18.1–22.4 (21.1 ± 1.6)	19.2–25.2 (22.0 ± 1.6)
PFL	15.1–18.9 (16.7 ± 1.0)	15.5–18.7 (16.9 ± 1.1)	14.5–17.1 (16.1 ± 0.9)	14.2–16.0 (15.3 ± 0.6)	15.3–18.5 (17.1 ± 1.0)	15.1–22.1 (19.3 ± 1.8)	13.5–19.4 (15.6 ± 1.7)
PFBL	3.2–4.3 (3.9 ± 0.3)	3.6–4.5 (4.0 ± 0.3)	3.4–4.5 (3.9 ± 0.4)	3.1–4.5 (3.9 ± 0.5)	4.0–5.6 (4.6 ± 0.5)	3.9–5.2 (4.5 ± 0.6)	2.7–4.5 (3.9 ± 0.5)
PVL	54.3–58.2 (56.1 ± 1.4)	48.6–52.2 (50.6 ± 1.0)	55.4–57.4 (56.5 ± 0.9)	53.8–55.8 (55.1 ± 0.8)	55.3–60.3 (56.3 ± 2.1)	52.2–56.4 (54.4 ± 1.7)	53.4–57.1 (55.1 ± 1.2)
VFL	11.1–13.6 (12.5 ± 0.8)	14.7–17.2 (15.8 ± 0.9)	14.5–17.1 (16.1 ± 0.9)	10.9–12.5 (11.8 ± 0.6)	11.9–14.7 (13.4 ± 0.9)	15.5–22.1 (19.3 ± 1.8)	12.1–15.7 (13.6 ± 1.2)
VFBL	3.0–4.0 (3.5 ± 0.4)	3.2–4.3 (3.8 ± 0.3)	3.0–4.2 (3.6 ± 0.4)	2.6–3.5 (3.2 ± 0.3)	3.1–3.9 (3.3 ± 0.2)	3.8–5.4 (4.5 ± 0.6)	2.7–4.0 (3.2 ± 0.4)
PAL	78.6–82.6 (80.0 ± 1.1)	74.6–77.9 (75.6 ± 1.0)	77.3–81.6 (80.6 ± 1.5)	78.4–81.2 (79.6 ± 0.9)	80.1–83.0 (81.3 ± 1.0)	77.6–80.5 (79.1 ± 1.0)	76.7–81.7 (79.9 ± 1.4)
AFL	14.3–17.7 (15.7 ± 1.1)	16.1–18.1 (16.9 ± 0.6)	14.2–16.1 (15.0 ± 0.6)	14.3–15.6 (14.6 ± 0.6)	14.0–16.8 (15.6 ± 0.8)	14.4–19.2 (17.8 ± 1.5)	14.9–17.2 (16.1 ± 1.0)
AFBL	6.8–8.1 (7.4 ± 0.5)	7.3–9.4 (8.4 ± 0.6)	6.7–7.5 (7.1 ± 0.3)	5.9–7.6 (6.8 ± 0.5)	6.6–7.3 (7.0 ± 0.3)	7.5–9.0 (8.1 ± 0.5)	7.1–11.0 (8.4 ± 1.2)
PANL	72.2–77.4 (74.1 ± 1.6)	78.6–82.8 (80.1 ± 1.2)	73.3–76.6 (74.6 ± 1.2)	71.7–74.2 (73.5 ± 0.9)	72.0–78.6 (74.1 ± 2.0)	71.2–76.1 (73.5 ± 1.6)	68.5–76.2 (73.8 ± 2.4)
CPL	11.2–15.5 (13.1 ± 1.3)	14.3–17.8 (15.7 ± 1.0)	11.5–13.4 (12.4 ± 0.6)	11.6–14.1 (12.2 ± 0.8)	10.1–12.5 (11.0 ± 0.8)	11.0–14.5 (12.6 ± 1.0)	10.2–14.3 (11.5 ± 1.2)
CPD	9.7–11.7 (10.6 ± 0.8)	10.0–11.6 (10.8 ± 0.5)	10.1–11.3 (10.8 ± 0.4)	9.5–11.3 (10.2 ± 0.6)	9.8–12.0 (10.7 ± 0.8)	12.2–14.8 (13.0 ± 0.9)	8.5–13.4 (10.9 ± 1.5)
Percentage of HL (%)							
ED	13.6–19.5 (15.9 ± 1.7)	11.2–15.2 (12.4 ± 1.2)	10.4–14.9 (12.9 ± 1.7)	9.2–13.5 (10.9 ± 1.3)	9.0–14.2 (11.9 ± 1.6)	11.1–19.7 (15.1 ± 2.3)	12.5–16.8 (14.7 ± 1.7)
IW	41.8–47.3 (43.9 ± 1.7)	34.6–44.6 (41.3 ± 2.5)	42.3–47.9 (44.9 ± 2.1)	44.1–51.9 (48.1 ± 2.7)	39.9–45.2 (42.2 ± 2.1)	41.1–49.9 (46.1 ± 2.5)	34.5–45.6 (40.3 ± 3.2)
STL	30.2–34.7 (32.8 ± 1.6)	37.7–43.3 (40.7 ± 1.8)	31.2–40.2 (36.0 ± 3.3)	30.3–40.5 (35.1 ± 3.3)	33.5–40.5 (36.4 ± 2.0)	34.4–40.9 (37.3 ± 2.4)	31.2–37.9 (34.0 ± 2.0)
DAN	29.6–36.4 (33.0 ± 2.2)	23.6–39.2 (29.7 ± 3.9)	30.6–36.5 (34.1 ± 1.9)	29.1–35.6 (32.5 ± 2.3)	31.4–35.1 (32.8 ± 1.4)	31.6–38.0 (35.0 ± 2.6)	26.4–37.1 (30.4 ± 2.9)
DPN	34.0–39.1 (37.2 ± 1.8)	33.8–38.9 (36.5 ± 1.5)	34.5–40.5 (37.7 ± 1.9)	34.0–37.8 (36.0 ± 1.3)	33.8–38.2 (36.0 ± 1.7)	37.6–42.8 (39.3 ± 1.6)	34.3–41.6 (36.7 ± 2.3)
DAPN	8.0–11.1 (9.6 ± 0.9)	6.3–10.6 (7.7 ± 1.3)	8.2–13.2 (10.6 ± 2.0)	7.5–11.9 (10.5 ± 1.4)	8.0–13.0 (9.9 ± 1.7)	10.1–12.3 (11.0 ± 0.7)	8.1–12.4 (10.3 ± 1.6)
MBL	43.3–55.9 (48.0 ± 4.4)	36.4–50.8 (43.3 ± 4.7)	52.9–61.7 (56.1 ± 2.7)	37.3–55.4 (45.5 ± 5.3)	41.5–55.6 (46.0 ± 4.5)	38.3–56.3 (48.7 ± 5.3)	33.9–55.9 (45.5 ± 8.0)
OSBL	50.0–69.6 (59.4 ± 6.6)	47.4–58.7 (54.4 ± 4.4)	64.8–71.6 (68.8 ± 2.6)	41.3–52.5 (48.2 ± 5.6)	43.9–60.7 (51.5 ± 6.4)	47.2–66.5 (57.4 ± 5.8)	39.2–61.4 (53.9 ± 7.1)
ISBL	25.5–48.5 (38.2 ± 8.6)	25.6–37.4 (33.1 ± 3.4)	30.6–51.5 (42.1 ± 6.4)	28.8–36.9 (33.1 ± 2.9)	28.7–40.8 (33.1 ± 4.6)	35.9–40.6 (37.8 ± 2.6)	21.1–47.9 (33.1 ± 7.4)
Percentage of CPL (%)							
CPD	67.2–98.7 (81.6 ± 8.9)	63.4–78.0 (68.7 ± 5.1)	75.1–97.5 (87.7 ± 6.8)	74.7–90.6 (83.3 ± 4.6)	95.2–118.2 (98.5 ± 10.1)	95.0–125.0 (103.4 ± 10.2)	73.8–131.0 (96.7 ± 20.5)
Habitat types	Small pools on surface where groundwater overflowed	Karst cave under mountain	Water outlet of underground karst cave	Open stream	Water of underground karst cave	Surface stream	Underground karst cave

**Table 2.** Uncorrected *p*-distances (%) between seven species in the genus *Oreonectes* based on mitochondrial Cyt *b* genes.

ID	Species	1	2	3	4	5	6
1	*Oreonectesandongensis* sp. nov.						
2	*Oreonectesdamingshanensis*	6.1					
3	*Oreonectesguananensis*	7.5	8.7				
4	*Oreonectesguilinensis*	7.0	7.2	8.8			
5	*Oreonectesluochengensis*	6.4	7.5	4.8	8.1		
6	*Oreonectesplatycephalus*	6.6	6.7	8.7	6.5	7.9	
7	*Oreonectespolystigmus*	6.0	6.0	8.3	7.3	7.4	6.4

Body elongated and cylindrical, deepest body depth in front of dorsal-fin origin, deepest body depth 16–18% of standard length (SL). Head slightly depressed and flattened, maximum head width greater than deepest head height. Anterior and posterior nostrils adjacent, distance shorter than diameter of posterior nostril. Base of anterior nostril tube-shaped with elongated barbel-like tip; barbel longer than anterior nostril tube. Eyes normal, eye diameter 14–20% of head length (HL). Snout obtuse, snout length shorter than postorbital length. Mouth inferior, lips smooth, center of lower lip with notch. Three pairs of barbels, inner rostral barbel length 23–49% of HL, extending to the anterior margin of eye; outer rostral barbel length 50–70% of HL, extending to the posterior margin of eye; maxillary barbel length 43–56% of HL, not reaching to posterior margin of opercula.

Dorsal-fin origin slightly posterior to pelvic-fin origin. Predorsal length 58–62% of SL. Tip of pectoral fin not reaching half of distance between origin of pectoral and pelvic fins. Tip of pelvic fin not reaching anus. Short distance (two times eye diameter) between anal-fin origin and anus. Caudal fin straight. Caudal peduncle without adipose crests along both dorsal and ventral sides. Caudal peduncle depth 67–99% of caudal peduncle length. Whole body covered by scales except head. Lateral line incomplete, with 8–16 pores. Cephalic lateral line system developed, with 6–9 supraorbital pores, 3 + 8–10 infraorbital pores, three or four canal pores, and 6–8 pre-operculo-mandibular canal pores.

Stomach U-shaped. Swim bladder divided into two chambers, anterior chamber covered by dumbbell-shaped bony capsule, and posterior chamber developed with posterior extremely reaching below dorsal-fin origin.

##### Coloration.

In formalin-fixed specimens, dorsal surface and trunk of body yellowish, while abdomen appears grayish. Additionally, dorsal surface and flank with small spots or short bars. Dorsal and caudal fins with black speckles. Longitudinal stripe extending from gill opening to caudal peduncle in female, lacking in males.

##### Sexual dimorphism.

In reproductive season, males possess large genital papilla located immediately posterior to anus, unclear in females; gonad opens at end of fleshy prominence.

##### Distribution and habitat.

*Oreonectesandongensis* sp. nov. was collected from Andong Township, Xincheng County, Laibin City, Guangxi Zhuang Autonomous Region, China, a tributary of the Hongshui River in Xijiang River basin. During the rainy season, specimens were gathered from small pools on the surface where groundwater had overflowed. *Troglonectescanlinensis*Li et al., 2023 specimens were also collected from the same pool.

##### Etymology.

The nomenclature of this species is derived from the Chinese pinyin of Andong, the name of the village where the specimens were obtained. We suggest the Chinese common name as “安东岭鳅”.

##### Remarks.

*Oreonectesandongensis* sp. nov. can be distinguished from *O.damingshanensis* by the six branched pelvic-fin rays (vs 7), a dorsal-fin origin slightly posterior to pelvic-fin origin (vs posterior to pelvic-fin origin obviously), and 11 or 12 inner gill rakers on the first gill arch (vs 9), from *O.guananensis* by six branched pelvic-fin rays (vs 7 or 8), dorsal-fin origin slightly posterior to pelvic-fin origin (vs opposite to pelvic-fin origin), caudal with irregular black markings (vs without irregular black markings), and maxillary barbel not reaching to the gill cover (vs reaching to the gill cover), from *O.guilinensis* by lateral line pores 8–16 (vs 4–6), tip of pelvic fin not reaching to anus (vs exceeding to anus), and maxillary barbel not reaching to the opercula (vs reaching to posterior margin of the eye), from *O.luochengensis* by cephalic lateral line system present (vs absent), abdomen between pectoral-fin origin to pelvic-fin origin scaled (vs scaleless), from *O.platycephalus* by posterior chamber of swim bladder developed (vs reduced), dorsal-fin origin slightly posterior to pelvic-fin origin (vs posterior to pelvic-fin origin obviously), seven branched dorsal-fin rays (vs 8 or 9), and six branched pelvic-fin rays (vs 8), from *O.polystigmus* by tip of pelvic fin not reaching to anus (vs exceeding to anus), and maxillary barbel not reaching to the opercula (vs reaching to the pectoral-fin origin).

### ﻿*Key to species of the genus*Oreonectes

**Table:** 

1	Body colorless	***O.luochengensis***
–	Color pattern present	**2**
2	Dorsal-fin origin opposite to pelvic-fin origin	***O.guananensis***
–	Dorsal-fin origin posterior to pelvic-fin origin	**3**
3	Tip of pelvic fin exceeding anus	**4**
–	Tip of pelvic fin not reaching anus	**5**
4	Six branched dorsal-fin rays	***O.guilinensis***
–	Seven branched dorsal-fin rays	***O.polystigmus***
5	Posterior chamber of swim bladder reduced	***O.platycephalus***
–	Posterior chamber of swim bladder developed	**6**
6	Six branched pelvic fin rays, 11 or 12 inner gill rakers on first gill arch	***Oreonectesandongensis* sp. nov.**
–	Seven branched pelvic fin rays, 9 inner gill rakers on first gill arch	***O.damingshanensis***

## ﻿Discussion

The distinct lineage of *Oreonectesandongensis* sp. nov. marked by an uncorrected *p*-distance of 6.0% from *O.polystigmus*, along with notable morphological differences, substantiates its validity as a new species. With the addition of the new species, the genus *Oreonectes* now comprises seven species: *Oreonectesandongensis* sp. nov., *O.damingshanensis*, *O.guananensis*, *O.guilinensis*, *O.luochengensis*, *O.platycephalus*, and *O.polystigmus*.

*Oreonectesandongensis* sp. nov. exhibited sexual dimorphism. Notably, males contained larger genital papilla located immediately posterior to the anus, with the gonads opening at the end of a fleshy prominence. Zhu (1989) noted that this structure may be related to the special breeding habits of *Oreonectes*. Additionally, most species of *Oreonectes* possess that developed posterior chamber of swim bladder except *O.platycephalus*. Zhu (1989) speculated that the posterior chamber of swim bladder related to adapting to habitat. *Oreonectesandongensis* sp. nov. and other congeneric species (except *O.platycephalus*) possess developed posterior chamber of swim bladder, and mainly habitat in pools surround subterrane river, but *O.platycephalus* inhabit in running water of upper reaches of the river.

Within the genus *Oreonectes*, *O.platycephalus* has a broader distribution, both within and beyond Guangxi, whereas the other species are exclusively found in Guangxi. In areas within Guangxi, *O.platycephalus* coexists with the other species, with their distribution overlapping along certain routes of *O.platycephalus* (Tang 2012). Wang (2022) suggested that the ancestors of the Nemacheilidae family in southwest China emerged around 26.19 million years ago (approximately the Late Oligocene). The uplift of the Tibetan Plateau led to the development of the Pearl River occurred concurrently (Zhang et al. 2022). This expansion towards the Pearl River Delta area likely facilitated the spread of *Oreonectes*. As the caves in southwest China continued to develop, some fish species entered these habitats, evolving traits suited to cave living, such as eye degeneration, loss of scales, and lack of pigmentation.

### ﻿Comparative material

All specimens were collected from Guangxi; their measurements are given in Appendix 2.

*Oreonectesguananensis*, KIZ2010003067, holotype, 71.5mm SL, KIZ2010003068–072, paratypes, 5 ex., 51.0–71.9 mm SL, Guan’an Village, Changmei Town, Huanjiang County, Guangxi.

*Oreonectesguilinensis*, ASIZB208001, holotype, 73.5 mm SL, ASIZB208002–007, paratypes, 6 ex., 52.0–68.3 mm, Shigumen Village Xingping Town, Yangshuo County, Guilin City, Guangxi.

*Oreonectesluochengensis*, KIZ2010003073, holotype, 71.2 mm SL, KIZ2010003074–077, KIZ2010003242–244, paratypes, 7 ex., 61.3–74.7 mm SL, Tianhe Town, Luocheng County, Guangxi.

*Oreonectesplatycephalus*, KIZ2003007105–106, 63.2–64.2 mm SL, KIZ2003007110, 60.9 mm SL, KIZ2005006211–212, 38.7–43.1 mm SL, KIZ2005006214–216, 45.6–47.7 mm SL, 8 ex., Fenzhan Village, Jinxiu County, Guangxi.

*Oreonectespolystigmus*, KIZ2001004626, holotype, 54.7 mm SL, KIZ2002004627–634, paratypes, 9 ex., 33.7–52.4 mm SL, Dabu Village, Guilin City, Guangxi.

## Supplementary Material

XML Treatment for Oreonectes andongensis

## ﻿Additional information

### Author ORCIDs

Xue-Ming Luo https://orcid.org/0000-0001-6441-4817

Li-Na Du https://orcid.org/0000-0002-2246-643X

### Data availability

All of the data that support the findings of this study are available in the main text or Supplementary Information.

## ﻿Appendix 1

**Table A1.** Localities, voucher information, and GenBank numbers for all samples used in this paper.

Species	Locality	Voucher	GenBank number
*Triplophysanasobarbatula*	Libo County, Guizhou, China	GZNU20190114001	ON116529
*Triplophysabaotianensis*	Nanpanjiang River, Panzhou City, Guizhou, China	GZNU20180421005	NC056365
*Triplophysazhenfengensis*	Xingren County, Guangxi, China	GZNU20180419002	NC063617
*Traccatichthyspulcher*	Daxin County, Guangxi, China	Tissue ID: GX1	ON116516
*Traccatichthyszispi*	Hainan, China	Tissue ID:HN01	ON116518
*Traccatichthystaeniatus*	N/A	N/A	NC031581
*Lefuanikkonis*	N/A	N/A	NC027662
*Lefuacostata*	N/A	N/A	NC029385
*Lefuaechigonia*	N/A	N/A	NC004696
*Oreonectesplatycephalus*	Shenzhen City, Guangdong, China	GZNU2020112501	ON116528
*Oreonectesguilinensis*	Xingping Town, Yangshuo County, Guangxi, China	N/A	MN239094
*Oreonectesdamingshanensis*	Leping Village, Guling Town, Mashan County, Guangxi, China	GZNU20230216011	OQ754117
*Oreonectesdamingshanensis*	Leping Village, Guling Town, Mashan County, Guangxi, China	GZNU20230216012	OQ754118
*Oreonectesdamingshanensis*	Damingshan Mountain, Shanglin County, Guangxi, China	GZNU 2020112502	ON116496
*Oreonectespolystigmus*	Dabu Town, Yanshan District, Guilin, Guangxi, China	GZNU2020011501	ON116514
*Oreonectesandongensis* sp. nov.	Andong Town, Xincheng County, Laibin City, Guangxi, China	N/A	OR188128
*Oreonectesandongensis* sp. nov.	Andong Town, Xincheng County, Laibin City, Guangxi, China	N/A	OR712240
*Oreonectesandongensis* sp. nov.	Andong Town, Xincheng County, Laibin City, Guangxi, China	N/A	OR712241
*Oreonectesluochengensis*	Tianhe Town, Luocheng County, Guangxi, China	GZNU2020011502	ON116495
*Oreonectesguananensis*	Changmei Town, Huanjiang County, Guangxi, China	GZNU2020073102	ON116507
*Guinemachiluslongibarbatus*	Hechi City, Guangxi, China	GZNU20210823001	ON148334
*Guinemachiluspseudopulcherrimus*	Dongmiao Village, Du’An County, Hechi City, Guangxi, China	D1925	OQ024375
*Micronemacheiluscruciatus*	N/A	N/A	NC033960
*Micronemacheiluspulcherrimus*	Duan County, Hechi City, Guangxi, China	GZNU20210609004	ON116493
*Karstsinnectesparva*	Ande Town, Jingxi City, Guangxi, China	Tissue ID: JTQ02	ON116520
*Karstsinnectesanophthalmus*	Leiping Town, Daxin County, Guangxi, China	GZNU2019011310	ON116513
*Karstsinnectesacridorsalis*	Bamu Town, Tiane County, Guangxi, China	GZNU2020	ON116515
*Troglonectestranslucens*	Xiaao Town, Duan County, Guangxi, China	GZNU 2020082302	ON116510
*Troglonectesmacrolepis*	Dacai Town, Huanjiang County, Guangxi, China	GZNU2019122202	NC073129
*Troglonectesmicrophthalmus*	Tianhe Town, Luocheng County, Guangxi, China	GZNU2020041601	NC073127
*Troglonectescanlinensis*	Andong Town, Xincheng County, Liuzhou City, Guangxi, China	c FFL-2023	OQ129618
*Troglonectesfurcocaudalis*	Yongle Town, Rongshui County, Guangxi, China	GZNU2020042701	ON116512
*Paranemachiluszhengbaoshani*	Duan County, Guangxi, China	GZNU20210526001	ON116530
*Paranemachiluschongzuo*	Daxin County, Chongzuo City, Guangxi, China	N/A	MW532082
*Paranemachilusjinxiensis*	N/A	N/A	NC039380
*Paranemachilusgenilepis*	N/A	N/A	MT845213
*Yunnanilusanalis*	N/A	N/A	MW729320
*Yunnaniluspleurotaenia*	N/A	QT95	MW192447
*Yunnanilusjiuchiensis*	Jiuchi County, Penzhou City, Sichuan, China	N/A	MW532080

## ﻿Appendix 2

**Table A2.** Measurements of the eight species of *Oreonectes*. All units are in mm.

Species	Voucher number	TL	SL	BD	BW	HL	HD	HW	ED	IW	STL	PDL	PPL	PVL	PAL	PANL	CPL	CPD	DFL	DFBL	PFL	PFBL	VFL	VFBL	AFL	AFBL	MBL	OSBL	ISBL	DAN	DPN	DAPN
*O.andongensis* sp. nov.	GXNU20220601	74.9	60.2	10.8	9.1	13.3	7.5	10.3	2.0	5.9	4.6	37.5	12.6	35.0	49.7	46.6	6.8	6.7	10.8	6.9	11.4	2.3	8.2	2.1	8.7	4.3	6.9	8.3	6.3	4.3	4.7	1.5
	GXNU20220602	68.7	56.4	10.0	7.5	12.7	7.0	8.8	2.1	5.3	3.9	35.0	10.9	32.4	46.2	42.1	7.0	6.6	10.7	5.8	9.1	2.3	7.1	2.2	8.7	4.4	7.0	7.9	5.7	4.6	4.8	1.2
	GXNU20220603	67.2	54.4	9.4	6.5	12.2	6.2	8.3	1.8	5.1	3.9	31.4	10.8	30.2	44.2	40.6	6.4	5.3	10.5	6.3	9.6	2.1	7.3	2.0	9.4	4.0	6.0	7.8	5.5	4.1	4.7	1.2
	GXNU20220604	61.4	50.9	8.0	6.7	10.8	5.8	7.8	1.6	5.1	3.6	29.7	10.6	27.6	41.3	36.9	6.5	5.2	9.3	5.3	8.2	2.2	5.7	2.0	7.4	3.9	4.7	7.3	5.2	3.6	4.2	1.1
	GXNU20220605	57.3	47.5	7.6	6.1	10.2	5.6	7.1	1.4	4.6	3.5	28.0	10.7	26.8	38.3	34.9	6.4	5.0	8.5	4.9	7.2	1.9	6.0	1.7	7.3	3.7	4.6	5.3	3.8	3.2	4.0	0.9
	GXNU20220606	52.3	40.8	6.8	5.2	8.9	5.2	6.5	1.4	3.9	3.1	23.7	8.8	22.4	32.5	29.4	4.9	3.7	7.1	4.2	6.8	1.4	4.9	1.4	5.8	2.8	3.9	4.9	2.3	2.8	3.4	0.8
	GXNU20220607	45.9	36.7	6.4	4.5	9.0	4.5	6.1	1.3	4.0	2.9	22.7	8.7	21.3	29.8	27.9	4.9	4.3	6.6	3.9	6.2	1.6	4.7	1.1	6.1	2.5	4.1	4.6	2.7	2.8	3.2	0.7
	GXNU20220608	49.6	40.4	6.4	4.7	9.0	4.8	5.9	1.8	3.8	2.9	24.0	8.5	22.2	32.4	29.3	5.4	4.4	6.8	3.9	6.7	1.7	4.6	1.2	6.4	3.2	4.0	5.5	2.4	3.2	3.5	0.9
	GXNU20220609	48.1	38.4	6.4	4.7	8.4	4.9	6.1	1.4	3.8	2.9	22.9	7.7	21.0	30.2	27.9	5.8	3.9	7.1	3.8	6.5	1.6	4.9	1.5	6.8	3.1	4.7	5.8	3.8	2.9	3.0	0.8
	GXNU20220610	46.1	36.5	5.9	4.2	8.5	4.5	5.6	1.5	3.7	2.6	22.4	8.0	20.7	29.6	27.3	5.7	4.1	6.8	3.3	6.0	1.2	4.7	1.1	5.6	2.5	4.0	4.2	2.8	2.5	2.9	0.9
*O.guananensis*	KIZ2010003067	90.2	71.5	11.8	8.7	16.6	8.3	11.3	1.8	7.4	5.2	41.6	15.7	41.0	58.3	52.8	9.6	7.2	12.7	6.8	11.6	2.8	8.8	3.0	10.5	5.3	9.1	11.3	7.8	5.1	5.7	1.4
	KIZ2010003068	76.8	61.4	10.1	7.1	13.9	7.2	9.7	1.8	6.4	4.4	36.0	13.5	35.3	49.6	47.1	7.8	6.9	11.0	6.0	10.5	2.3	8.0	2.2	9.5	4.5	7.6	9.8	5.9	4.8	5.0	1.8
	KIZ2010003069	69.6	56.3	9.9	5.3	12.3	6.3	8.3	1.7	5.9	5.0	33.5	12.2	31.2	45.7	41.3	6.8	6.1	10.0	5.4	8.9	2.1	7.6	1.9	8.5	4.0	7.6	8.8	6.3	4.3	4.7	1.2
	KIZ2010003070	66.2	53.3	10.5	5.2	12.3	6.4	9.0	1.8	5.2	4.6	30.5	11.8	29.7	43.5	39.9	6.4	5.7	9.7	5.8	8.6	2.3	6.7	1.7	7.6	3.6	6.9	7.9	5.0	4.1	4.8	1.6
	KIZ2010003071	62.9	51.0	9.7	6.1	11.2	5.8	8.4	1.6	5.2	4.2	29.3	10.9	28.3	39.4	37.7	6.5	5.5	9.5	5.6	8.7	1.7	6.3	2.1	8.2	3.8	5.9	7.4	3.4	4.1	4.5	0.9
	KIZ2010003072	88.5	71.9	11.7	9.4	16.6	7.8	11.3	1.7	7.0	6.2	42.6	16.6	41.3	58.5	54.4	8.3	8.1	12.7	7.0	10.5	3.2	10.1	2.2	10.5	4.8	9.2	11.8	6.8	5.9	6.2	1.8
*O.guilinensis*	ASIZB208001	89.7	73.5	12.3	9.3	16.0	10.3	12.2	1.6	7.8	6.1	43.4	38.6	39.9	39.7	40.0	31.4	38.3	12.2	6.8	10.7	2.7	9.0	2.2	10.9	4.3	7.1	7.4	4.6	5.7	6.0	1.9
	ASIZB208002	81.4	67.3	12.3	9.1	14.1	8.1	10.7	1.3	7.0	5.0	15.0	12.9	13.8	12.0	11.4	10.2	12.6	12.8	5.9	10.5	2.9	8.4	2.3	10.1	5.1	6.9	7.4	5.2	4.1	4.8	1.5
	ASIZB208003	82.8	68.3	12.4	10.1	14.8	9.1	12.1	2.0	7.5	6.0	40.4	37.0	38.1	37.5	36.4	29.1	35.4	11.6	6.3	10.9	3.1	8.3	2.2	9.8	4.8	8.2	6.7	4.4	5.1	5.6	1.7
	ASIZB208004	83.8	67.1	12.4	9.7	14.5	9.0	11.7	1.5	6.7	4.4	59.7	54.2	53.7	53.2	53.0	41.3	52.3	12.0	4.8	10.3	2.1	7.3	2.3	9.8	4.2	6.0	7.7	5.1	4.8	5.0	1.7
	ASIZB208005	82.2	67.6	12.4	9.3	14.3	8.2	10.4	1.7	6.3	4.5	54.4	49.8	49.0	48.7	49.7	38.6	48.6	11.5	5.5	9.6	2.9	7.9	2.2	9.7	4.6	6.4	5.9	4.5	4.9	5.1	1.4
	ASIZB208006	63.2	52.0	9.5	7.4	12.6	7.9	9.9	1.3	5.7	4.4	8.8	7.8	8.5	7.9	9.5	6.3	7.6	8.7	5.4	8.0	2.0	6.3	1.8	8.1	3.8	4.7	5.3	4.4	3.8	4.5	1.3
	ASIZB208007	79.6	65.5	11.8	9.1	13.3	8.6	11.0	1.5	6.9	4.6	7.3	6.4	7.7	6.9	7.1	5.1	6.4	11.4	5.6	10.5	2.2	7.2	1.7	8.8	4.5	6.1	7.6	4.6	4.1	4.9	1.0
*O.luochengensis*	KIZ2010003073	86.0	71.2	11.4	7.4	14.7	11.6	10.7	2.1	6.0	5.5	42.5	14.4	39.4	58.2	52.8	7.3	8.6	13.0	7.5	10.9	2.9	8.5	2.2	11.4	5.0	6.1	7.3	4.4	5.0	5.6	1.4
	KIZ2010003074	83.9	67.0	11.5	6.6	15.5	12.0	9.2	2.0	6.2	5.2	39.6	13.2	36.4	53.7	48.7	6.9	6.6	12.7	6.7	11.7	2.8	9.0	2.3	10.9	4.4	6.7	7.7	4.5	4.9	5.3	1.3
	KIZ2010003075	86.3	68.8	11.8	7.8	15.3	12.5	10.4	1.8	6.7	5.8	41.8	14.2	38.4	55.7	50.3	7.8	7.5	13.2	6.9	12.7	3.1	10.1	2.4	11.6	4.9	8.5	9.3	6.2	5.2	5.8	1.2
	KIZ2010003076	87.0	71.2	13.0	8.6	15.9	12.4	10.0	1.6	6.9	5.5	42.4	14.3	39.7	57.2	51.6	7.4	7.5	12.3	6.8	11.9	2.9	9.3	2.3	10.9	5.1	7.2	8.3	4.6	5.0	5.4	1.5
	KIZ2010003077	77.2	62.2	10.7	6.7	15.1	11.7	10.5	2.0	6.7	5.4	37.8	13.6	37.5	51.3	48.9	7.0	7.1	11.4	6.3	10.8	3.0	8.2	1.9	9.6	4.6	7.5	9.4	5.6	5.0	5.2	2.0
	KIZ2010003242	84.9	68.6	12.6	6.9	15.3	11.6	10.1	1.8	6.2	5.5	40.2	13.1	37.2	55.3	49.4	6.9	6.8	12.7	6.6	11.6	3.0	9.8	2.7	10.5	5.0	6.4	6.7	4.8	4.9	5.4	1.3
	KIZ2010003243	92.1	74.7	12.9	8.9	16.6	13.1	11.5	1.5	7.5	6.1	47.2	15.2	44.4	62.0	55.5	8.7	8.7	12.6	7.3	12.0	3.6	9.3	2.4	10.5	4.9	7.2	7.4	4.8	5.2	6.0	2.1
	KIZ2010003244	76.4	61.3	10.3	6.2	13.3	10.9	9.1	1.6	5.4	5.4	35.5	12.4	33.9	49.6	46.2	7.6	6.0	11.3	6.0	11.3	3.4	8.6	2.0	9.6	4.2	6.4	6.6	5.1	4.7	5.0	1.4
*O.platycephalus*	KIZ2003007105	82.5	64.2	12.4	6.8	16.3	12.5	11.2	2.4	6.7	6.1	41.5	14.1	36.2	51.3	48.8	7.1	8.9	13.6	6.8	12.9	3.5	11.5	2.7	12.1	4.9	9.2	10.9	6.3	5.7	6.2	1.8
	KIZ2003007106	80.1	63.2	12.0	6.0	13.2	11.0	9.9	1.9	5.9	4.6	41.1	14.4	35.7	50.6	46.5	7.7	7.9	12.0	6.4	11.5	2.5	9.7	2.1	10.5	5.2	6.9	8.2	5.3	4.9	5.1	1.6
	KIZ2003007110	75.9	60.9	10.9	6.2	13.0	10.7	9.7	1.4	5.9	5.0	38.3	11.6	34.1	47.2	45.7	7.8	7.5	9.5	6.0	9.4	2.5	8.8	2.2	8.8	4.6	6.5	7.0	4.2	4.0	4.9	1.5
	KIZ2005006211	56.7	43.1	7.2	4.6	10.5	8.6	7.0	1.5	5.0	4.3	26.7	9.6	22.8	34.5	31.7	6.3	5.8	8.5	4.1	8.5	1.9	7.6	1.6	8.2	3.7	5.4	5.5	4.2	4.0	4.3	1.2
	KIZ2005006212	49.6	38.7	6.2	4.5	9.0	7.5	6.1	1.8	4.0	3.3	23.3	8.5	21.6	30.4	28.8	5.1	5.7	8.0	4.3	8.5	2.0	7.2	1.8	7.4	3.5	4.6	5.6	3.4	3.0	3.4	0.9
	KIZ2005006214	56.9	46.5	6.9	4.7	10.4	8.5	7.2	1.7	5.0	4.2	28.4	8.4	24.6	36.3	33.3	5.6	5.8	9.5	4.6	9.1	1.8	7.7	1.8	8.4	3.7	4.8	4.9	4.1	3.9	4.1	1.1
	KIZ2005006215	61.6	47.7	7.8	5.1	10.9	9.4	7.0	1.7	5.0	3.7	28.6	10.5	25.2	38.4	34.6	6.4	6.1	9.4	4.6	9.1	2.3	8.0	2.0	8.8	3.7	4.7	6.1	3.9	4.0	4.6	1.3
	KIZ2005006216	58.0	45.6	7.8	4.8	10.0	8.2	6.7	1.5	5.0	3.5	27.4	9.2	23.8	35.9	32.5	5.5	5.5	8.8	4.5	9.3	2.1	8.0	1.8	8.2	3.8	3.8	5.8	3.7	3.2	4.0	1.0
*O.polystigmus*	KIZ2001004626	67.8	54.7	10.5	6.5	12.9	7.2	9.6	1.7	5.9	4.5	33.1	11.6	30.9	44.1	39.9	5.6	7.3	10.1	6.0	8.3	2.0	7.0	1.7	8.6	4.3	6.5	7.9	4.7	4.8	5.4	1.1
	KIZ2001004627	62.7	52.4	6.6	3.9	11.9	5.9	7.7	1.5	4.8	4.3	31.4	11.5	28.8	41.7	39.2	6.1	5.8	8.9	4.8	7.1	2.0	6.3	1.6	8.1	3.9	6.4	5.4	3.5	3.4	4.4	1.0
*O.polystigmus*	KIZ2001004628	45.8	37.4	5.3	2.9	8.9	4.2	5.1	1.2	3.3	2.9	23.9	7.2	20.4	30.6	28.5	4.3	3.2	6.8	3.7	6.2	1.6	5.9	1.4	6.1	4.1	3.0	4.6	3.2	2.7	3.3	1.1
	KIZ2001004629	51.9	42.5	7.5	3.2	9.5	5.1	5.9	1.6	3.8	3.3	25.6	9.2	22.9	32.5	29.1	6.1	4.2	7.5	4.2	7.0	1.8	5.2	1.1	6.8	3.6	4.9	5.5	3.1	2.9	3.3	1.2
	KIZ2001004630	41.8	33.7	6.6	3.1	8.2	4.3	5.2	1.4	3.6	2.8	19.9	8.5	18.8	27.4	25.0	3.5	3.9	6.7	3.6	5.3	1.5	4.9	1.0	5.0	2.6	2.9	4.6	1.7	2.7	3.2	0.7
	KIZ2001004631	44.5	36.0	5.8	3.0	8.6	4.6	5.9	1.4	3.4	3.3	21.8	8.3	20.6	28.9	27.5	4.0	4.3	6.1	3.2	5.2	1.5	4.5	1.4	5.4	2.6	3.5	4.9	2.7	2.3	3.0	1.1
	KIZ2001004632	49.5	39.9	6.9	3.5	9.6	5.1	6.2	1.2	3.8	3.0	25.5	9.3	21.3	32.0	30.1	4.7	3.8	7.1	3.6	5.6	1.1	5.9	1.1	6.9	3.9	3.7	3.8	2.4	2.8	3.5	0.9
	KIZ2001004633	51.0	41.2	6.6	3.7	9.9	4.9	6.3	1.6	3.4	3.4	24.5	8.5	22.7	32.8	29.2	4.2	5.0	8.1	4.0	8.0	1.7	5.9	1.2	7.5	3.3	5.0	6.1	4.7	3.0	3.5	1.0
	KIZ2001004634	55.2	44.4	6.6	4.0	10.5	5.6	7.4	1.5	4.5	3.3	26.8	10.0	24.4	35.3	33.4	5.2	4.3	7.8	5.4	7.0	1.9	6.0	1.6	7.4	4.0	5.9	5.8	4.1	3.0	3.6	1.1
